# Astragaloside IV, a Novel Antioxidant, Prevents Glucose-Induced Podocyte Apoptosis *In Vitro* and *In Vivo*

**DOI:** 10.1371/journal.pone.0039824

**Published:** 2012-06-22

**Authors:** Dingkun Gui, Yongping Guo, Feng Wang, Wei Liu, Jianguo Chen, Yifang Chen, Jianhua Huang, Niansong Wang

**Affiliations:** 1 Department of Nephrology, Zhejiang Hospital, Hangzhou, China; 2 Department of Nephrology and Rheumatology, Sixth People's Hospital Affiliated to Shanghai Jiao Tong University, Shanghai, China; 3 Department of Gastroenterology, No 1 Hospital of Chenzhou, Chenzhou, China; 4 Institute of Integrated Chinese and Western medicine, Huashan Hospital, Fudan University, Shanghai, China; Wayne State University, United States of America

## Abstract

Glucose-induced reactive oxygen species (ROS) production initiates podocyte apoptosis, which represents a novel early mechanism leading to diabetic nephropathy (DN). Here, we tested the hypothesis that Astragaloside IV(AS-IV) exerts antioxidant and antiapoptotic effects on podocytes under diabetic conditions. Apoptosis, albuminuria, ROS generation, caspase-3 activity and cleavage, as well as Bax and Bcl-2 mRNA and protein expression were measured in vitro and in vivo. Cultured podocytes were exposed to high glucose (HG) with 50, 100 and 200 µg/ml of AS-IV for 24 h. AS-IV significantly attenuated HG-induced podocyte apoptosis and ROS production. This antiapoptotic effect was associated with restoration of Bax and Bcl-2 expression, as well as inhibition of caspase-3 activation and overexpression. In streptozotocin (STZ)-induced diabetic rats, severe hyperglycemia and albuminuria were developed. Increased apoptosis, Bax expression, caspase-3 activity and cleavage while decreased Bcl-2 expression were detected in diabetic rats. However, pretreatment with AS-IV (2.5, 5, 10 mg·kg^−1^·d^−1^) for 14 weeks ameliorated podocyte apoptosis, caspase-3 activation, renal histopathology, podocyte foot process effacement, albuminuria and oxidative stress. Expression of Bax and Bcl-2 mRNA and protein in kidney cortex was partially restored by AS-IV pretreatment. These findings suggested AS-IV, a novel antioxidant, to prevent Glucose-Induced podocyte apoptosis partly through restoring the balance of Bax and Bcl-2 expression and inhibiting caspase-3 activation.

## Introduction

Diabetic nephropathy (DN) is an important and common complication of both type 1 and type 2 diabetes leading to end-stage renal disease (ESRD). Podocytes are terminally differentiated cells reside on the outer surface of the glomerular basement membrane (GBM) and play a key role in maintaining the structure and function of the glomerular filtration barrier. There is common agreement that podocytes have a central role in the development of proteinuria, which is a hallmark of almost all glomerular diseases [1]. In human type 1 and type 2 diabetes mellitus [2]–[4], the podocyte number is reduced not only in individuals with DN, but also in subjects with short duration of diabetes before the onset of microalbuminuria [4], [5]. Studies in type 1 and type 2 diabetes also demonstrated that podocyte depletion and loss represented one of the earliest mechanisms in the pathogenesis of DN [6], [7]. High glucose (HG) induced podocyte apoptosis in vitro and in vivo [7], and podocyte apoptosis contributed to podocyte loss and reduced podocyte number [8]. The study demonstrated that podocyte apoptosis coincided with the onset of urinary albumin excretion and preceded apparent podocyte loss in experimental diabetic models [7]. These studies indicate that DN may directly stem from podocyte injury. Thus, podocytes become a promising target for the development of new renal-protective drugs for DN.

The increased production of ROS played a critical role in the pathogenesis of DN. HG induced ROS production and accumulation of H_2_O_2_ in podocytes [9]. Furthermore, glucose-induced ROS production initiated podocyte apoptosis, which represented a novel early pathomechanism leading to DN in experimental type 1 and type 2 diabetic models [7]. Recent study futher confirmed that production of ROS by CYP4A monooxygenases, 20-HETE, and Nox oxidases was involved in apoptosis of podocytes in vitro and in vivo [10]. These results strongly suggest that ROS-mediated podocyte apoptosis is a novel early mechanism leading to DN. Thus, the concept of ROS-mediated podocyte apoptosis, associated with hyperglycemia, puts forth new and important opportunities to effectively prevent the development and progression of DN. However, there are no current interventions for DN specifically preventing podocyte apoptosis.

Astragaloside IV(AS-IV) was a saponin purified from Astragalus membranaceus (Fisch) Bge, which has been widely used in traditional Chinese medicine to treat renal diseases for a long time [11], [12]. It has been reported that AS-IV has an antioxidant effect and can ameliorate ischemia reperfusion (IR)-induced injury in brain [13] and heart [14]. However, the protective effects of AS-IV on apoptosis and oxidative stress in diabetic podocytes have not been investigated yet. We previously reported that AS-IV attenuated HG-induced podocyte detachment [15]. The aim of this study is to further examine the preventive effects of AS-IV on glucose-induced oxidative stress and podocyte apoptosis in vitro and in vivo, and then provide a novel drug therapy for the prevention of DN.

## Materials and Methods

### Drug preparation

Astragaloside IV(AS-IV) was purchased from Xi'an Sobeo Pharmaceutical Technology Company, Limited (purity above 98%, Xi'an, China). The chemical structure of Astragaloside IV (C_41_H_68_O_14_, molecular weight = 784) was described in our previous study [15].

### In vitro studies: Cell culture and AS-IV treatments

Conditionally immortalized mouse podocytes were kindly provided by Dr. Peter Mundel (Division of Nephrology, Massachusetts General Hospital, Harvard University) and were conducted as previously described [16]. The cells have a temperature-sensitive variant of the SV40 large T antigen (tsA58) that is induced by interferon-γ (IFN-γ). The cells proliferate at 33°C with IFN-γ, whereas the temperature 37°C without IFN-γ shifts the cells to differentiation. In brief, podocytes were maintained in RPMI 1640 supplemented with 10% heat-inactivated fetal calf serum (FCS, Gibco, USA), 100 U/ml penicillin and 100 µg/ml streptomycin. Cells were grown at 33°C with 10 U/ml mouse recombinant interferon-γ (IFN-γ, Sigma Chemical Corporation, USA). At confluence podocytes were incubated at 37°C on collagen-coated dishes for 14 days deprived of IFN-γ to allow differentiation. Differentiated podocytes were cultured for 24 h in RPMI 1640 medium and 1% FCS before being exposed to various experimental conditions. AS-IV was dissolved in dimethylsulfoxide (DMSO) and the final DMSO concentration did not exceed 0.1% (v/v). The cells were divided into the following groups: (1) normal glucose group (NG) as controls incubated in RPMI 1640 containing 5 mM glucose, (2) high glucose group (HG) incubated in RPMI 1640 containing 30 mM glucose, (3) mannitol group (MA) incubated in NG medium supplemented with 25 mM D-mannitol (Sigma, USA) as an osmotic control, (4) AS-IV group incubated in HG medium treated with different concentrations of AS-IV(50, 100, 200 µg/ml) for 24 h. All the glucose used in the present study was D-glucose. All experimental groups were cultured in quadruplicate.

**Figure 1:**
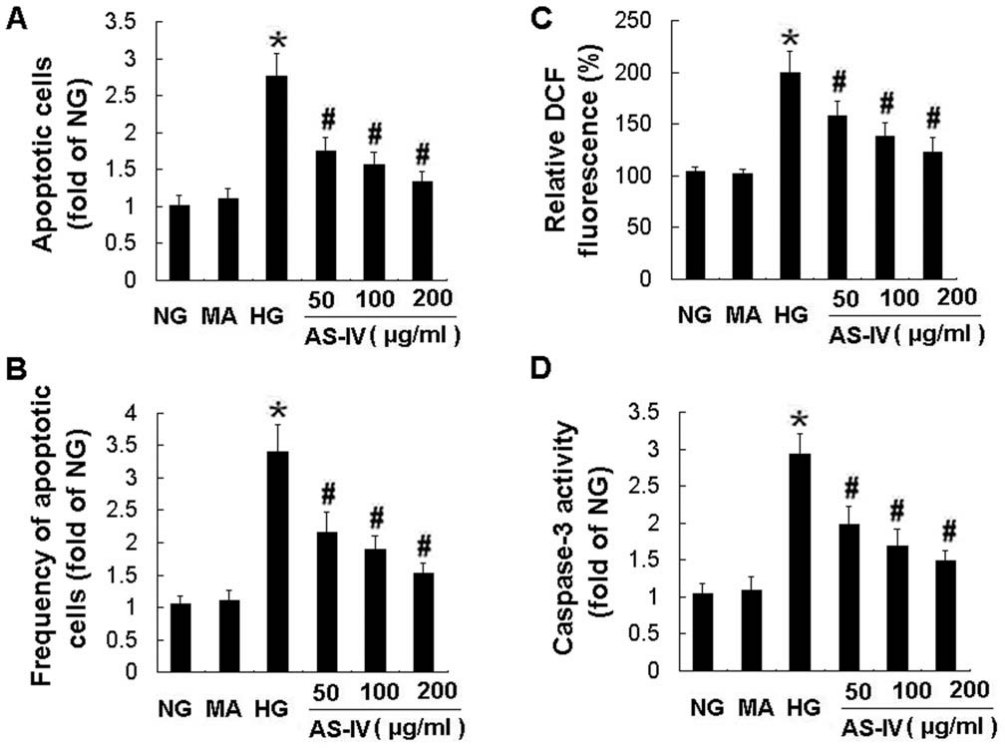
AS-IV dose-dependently inhibited HG-induced podocyte apoptosis, ROS production and caspase-3 activation. Effects of AS-IV on apoptosis assessed by Hoechst staining (A) and FACS (B), ROS production (C) and caspase-3 activity (D). Podocytes were exposed to NG, MA, HG, HG with 50, 100, 200 µg/ml of AS-IV for 24 h, respectively. NG, normal glucose (5 mM glucose); MA, mannitol (25 mM D-mannitol); HG, high glucose (30 mM glucose). Results are expressed as the means ± SD. *P<0.05 vs NG. ^#^ *P*<0.05 vs HG.

**Figure 2:**
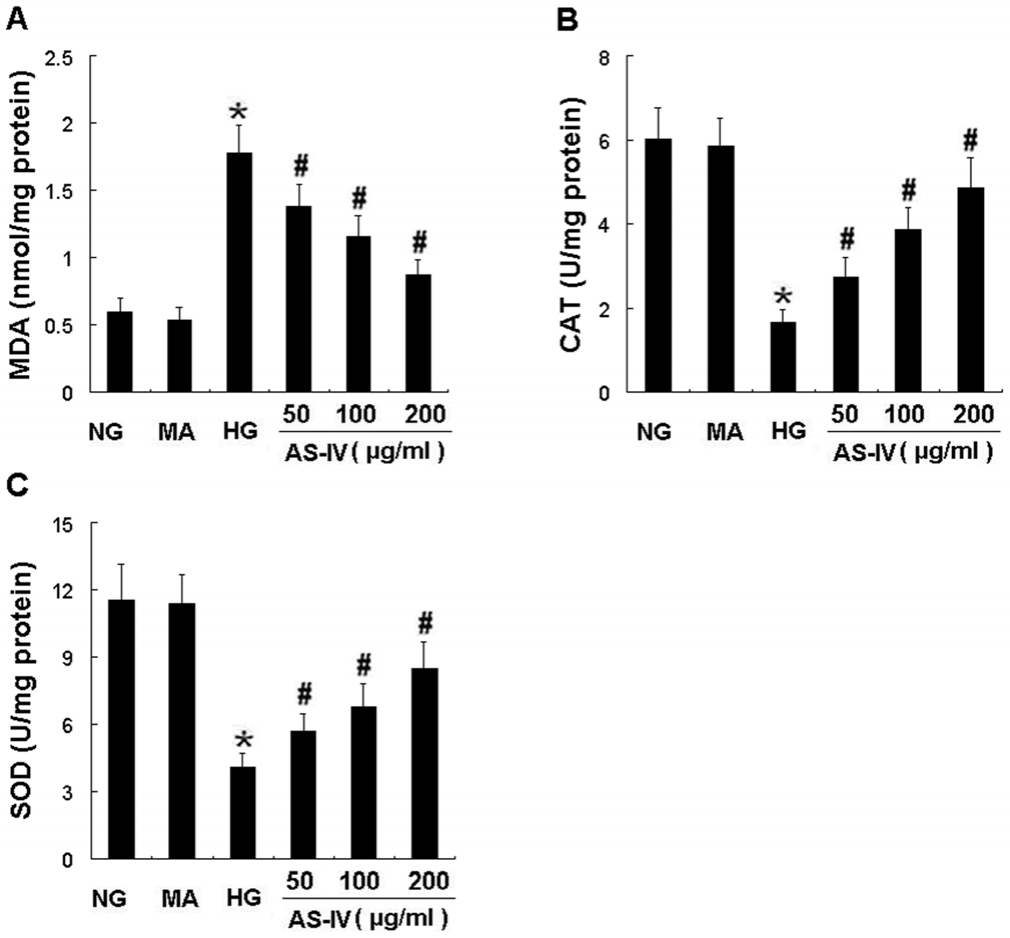
AS-IV dose-dependently lowered MDA content while elevated activities of CAT and SOD in HG-stimulated podocytes. Effects of AS-IV on content of MDA (A) and activities of CAT (B) and SOD (C) in podocytes. MDA, malondialdehyde; CAT, catalase; SOD, superoxide dismutase. Results are expressed as the means ± SD. *P<0.05 vs NG. ^#^ *P*<0.05 vs HG.

### In vivo studies: animal study and experimental design

All work with rat was approved by the Animal Ethics Committee of Fudan University, Shanghai, China and was performed in accordance with the “Guide for the Care and Use of Laboratory Animals” published by the National Institutes of Health. Healthy male Sprague-Dawley rats weighing 180 to 200 g, purchased from Experimental Animal Center, Fudan University, Shanghai, China, were housed in an air-conditioned room at 23±1°C with alternating 12 h cycles of light and dark. Animals were fed a standard diet and given water ad libitum. Diabetes was induced by a single intraperitoneal injection of streptozotocin (STZ) at 65 mg/kg in rats. Age-matched control rats received an equal volume of vehicle (0.1 M citrate buffer, pH 4.5). Forty-eight hours after injection of STZ, the blood glucose level was measured from the tail vein. Rats with a blood glucose level over 300 mg/dl were considered as diabetic rats. To investigate the preventive effects of AS-IV, AS-IV treatment (2.5, 5, 10 mg·kg^−1^·d^−1^) was started 2 weeks before STZ injection and lasted 14 weeks. The animals were divided into five groups (n = 8/each group): (1) Normal Sprague-Dawley rats as control (NC), (2) STZ-induced diabetic rats (DN), (3) STZ-induced diabetic rats treated with low dose of AS-IV at 2.5 mg·kg^−1^·d^−1^ (AL), (4) STZ-induced diabetic rats treated with moderate dose of AS-IV at 5 mg·kg^−1^·d^−1^ (AM) and (5) STZ-induced diabetic rats treated with high dose of AS-IV at 10 mg·kg^−1^·d^−1^ (AH). AS-IV was suspended in 1% carboxymethyl cellulose (CMC) solution at different concentrations and was given to the DN rats by oral gavage once daily. Normal rats (NC) received the same volume of CMC as control. Rats were kept in individual metabolic cages for 24 h urine collection at the end of 4, 8 and 12 weeks after STZ injection. Urine was centrifuged at 800 g for 10 min at 25°C. Whole urine was stored at −70°C and thawed just before use. Urinary albumin excretion (UAE) was measured using an ELISA Kit (Nanjing Jiancheng Bioengineering Institute, Nanjing, China) according to the manufacturer's method. At the end of 12 week after STZ, blood samples obtained from the tail vein were assayed for blood glucose and HbA1c levels. At the end of the study, rats were anesthetized with pentobarbital sodium and the blood samples were taken through the abdominal aorta for measuring biochemical parameters, including blood urea nitrogen (BUN), creatinine (Cr) and alanine aminotransferase (ALT), by an automatic biochemistry analyzer (Hitachi Model 7600, Japan). Animals were then killed and the kidneys were harvested immediately. The cortex from transversely bisected left kidneys was snap-frozen in liquid nitrogen and stored at −70°C for protein and total RNA extraction. That of the right kidneys was fixed with 10% buffered formalin and embedded in paraffin for histological evaluation. The kidneys were cut into 4 µm sections and stained with hematoxylin and eosin. The sections were then examined by light microscopy.

**Figure 3:**
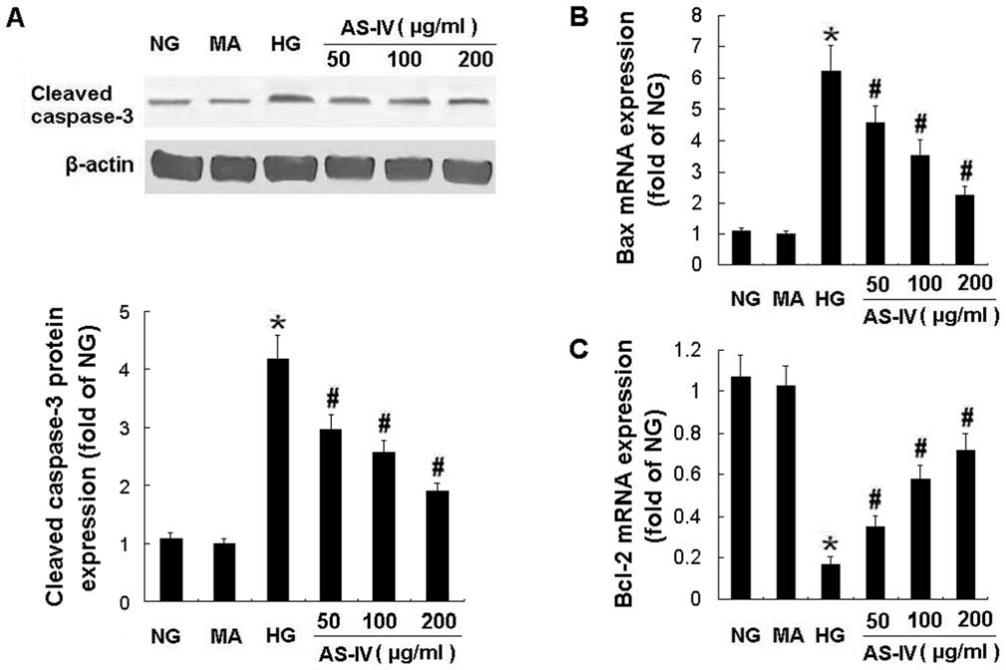
AS-IV decreased Bax mRNA and caspase-3 protein expression while increased Bcl-2 mRNA expression in podoctyes. Effects of AS-IV on cleaved caspase-3 protein expression (A), and mRNA expression of Bax (B) and Bcl-2 (C) in HG-stimulated podocytes. The Bax and Bcl-2 mRNA expression was examined by Real-time RT-PCR. Results are expressed as the means ± SD. *P<0.05 vs NG. ^#^ *P*<0.05 vs HG.

**Figure 4:**
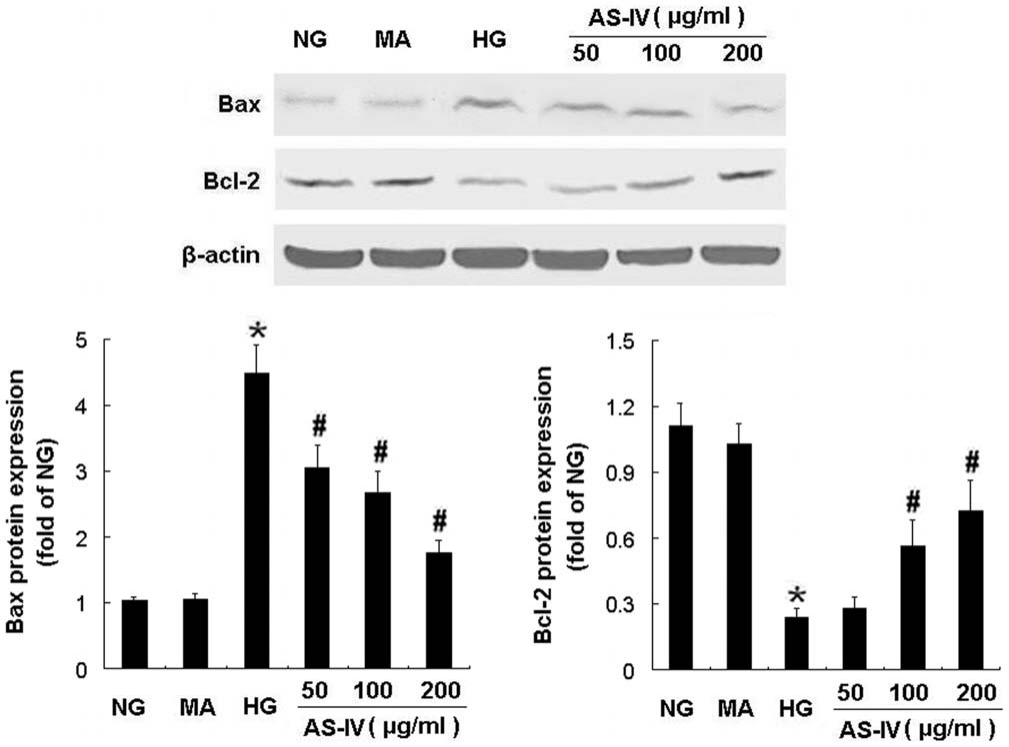
AS-IV downregulated Bax protein expression and upregulated Bcl-2 protein expression in podoctyes. Effects of AS-IV on protein expression of Bax and Bcl-2 in HG-stimulated podocytes. The protein expression of Bax and Bcl-2 was examined by Western blotting. Results are expressed as the means ± SD. *P<0.05 vs NG. ^#^ *P*<0.05 vs HG.

### Electron microscopy studies

To determine the structural changes in podocyte morphology, electron microscopic morphometric evaluation was carried out performed by routine procedures. Renal cortex samples were cut into 1 mm^3^ pieces on ice, immediately fixed in 2.5% glutaraldehyde, and then embedded. Ultrathin sections were examined by electron microscopy. All electron microscope photomicrographs were evaluated in a blind fashion.

### Apoptosis assay

Apoptotic nuclei were detected using Hoechst 33258 staining. Podocytes were exposed to the different experimental conditions as indicated above. Cells were fixed with 4% paraformaldehyde for 10 min at room temperature and stained by Hoechst 33258 (Sigma, USA) for 30 min at room temperature. After that, cells were washed twice using PBS for 3 min. Apoptosis was identified by nuclear condensation and/or fragmentation. The percentage of apoptotic cells was counted on using fluorescent microscopy.

The apoptotic podocytes were also quantified by fluorescence-activated cell sorting (FACS) scan after Annexin V-fluorescein isothiocyanate (FITC) and propidium iodide (PI) labeling as described in the manufacturer's protocol (Roche). After 24 h incubation with the indicated stimuli, cultured podocytes were washed twice using cold phosphate-buffered saline (PBS) on ice, trypsinized, and pelleted by centrifugation at 200 g for 5 min. The pellet was washed twice with PBS and resuspended in binding buffer. After that, cells were stained with Annexin V-FITC and PI. The stained cells were then analyzed for apoptosis using FACS scan. Cells positive for Annexin V–FITC and negative for PI were considered apoptotic.

**Table 1:** Metabolic and plasma biochemical parameters.

Group	BG (mg/dl)	HbA1c (%)	ALT(U/L)	BUN (mmol/L)	SCr (µmol/L)
NC	175.1±24.1	4.29±0.13	60.6±5.6	5.48±0.50	29.2±5.8
DN	624.4±75.1*	9.79±0.56*	62.6±7.1	6.14±0.47	33.8±5.2
AL	605.0±50.7*	9.50±0.64*	64.5±5.5	5.76±0.80	32.0±4.7
AM	586.6±64.7*	9.37±0.70*	63.9±3.4	5.98±0.41	31.2±6.1
AH	573.1±45.7*	9.27±0.45*	66.1±7.1	5.66±0.39	28.4±6.8

BG, blood glucose; HbA1c, hemoglobin A1c; BUN, blood urea nitrogen; SCr, serum creatinine; ALT, alanine aminotransferase. AS-IV treatment was started 2 weeks before STZ injection and lasted 14 weeks. NC, normal control rats; DN, STZ-induced diabetic rats; AL, DN rats treated with AS-IV (2.5 mg·kg^−1^·d^−1^); AM, DN rats treated with AS-IV (5 mg·kg^−1^·d^−1^); AH, DN rats treated with AS-IV (10 mg·kg^−1^·d^−1^).

* *P*<0.05 vs NC group.

A transferase-mediated dUTP nick-end labeling (TUNEL) assay was performed to detect apoptotic nuclei in kidney sections [8], [17]. TUNEL assay was conducted using a TUNEL detection kit (Roche Diagnostics, Mannheim, Germany) according to the manufacturer's instruction. Apoptosis was measured by the pathologist who was blinded to the treatment animals had received. A minimum of five fields per slide and six slides per group were counted by this method. Positive cells were counted as podocytes when residing on the outer surface of GBM. Cells residing in areas circumscribed by GBM were counted as endocapillary or mesangial cells. Apoptotic cells with nuclei staining dark brown were counted by light microscopy. For quantitative analysis, TUNEL-positive cells were quantified by counting five different fields at 400× magnification with two independent investigators who was blinded to the treatment that the animals had received.

### Caspase-3 Activity Assay

Caspase-3 activity in lysates from cultured podocytes and kidney cortex was determined using the Caspase-3 Colorimetric Assay Kit (Nanjing KeyGEN Biotech. Co., Ltd. Nanjing, China), according to the manufacturer's protocol. The protein concentration was measured by the Bradford method. Caspase-3 activity was expressed in terms of absorbance units (OD 405 nm) per milligram of protein.

**Table 2:** Physical parameter and renal oxidative stress markers.

Group	BW (g)	MDA (nmol/mg pro)	CAT (U/mg pro)	SOD(U/mg pro)
NC	523.1±37.9	16.9±1.8	25.2±3.2	68.5±7.0
DN	231.3±23.0*	37.0±4.3*	12.2±1.9*	38.8±3.1*
AL	249.0±35.8*	28.0±3.2#	16.2±2.2#	51.1±2.8#
AM	240.0±28.3*	23.8±2.4#	18.6±2.5#	55.3±3.5#
AH	243.8±33.6*	20.1±3.1#	22.6±2.8#	68.3±7.7#

BW, body weight; MDA, malondialdehyde; CAT, catalase; SOD, superoxide dismutase.

* *P*<0.05 vs NC group.

# *P*<0.05 vs DN group.

**Figure 5:**
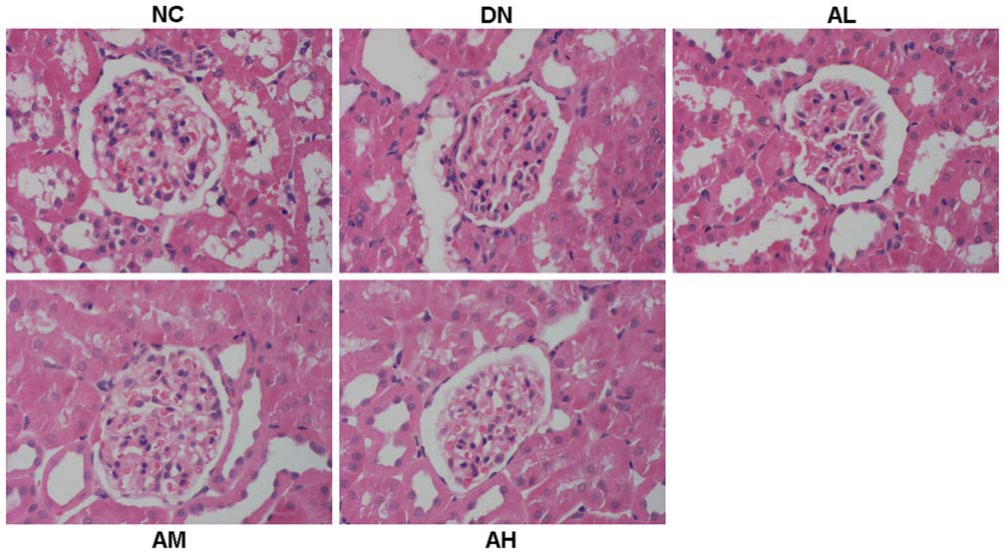
AS-IV ameliorated the renal histopathology in diabetic rats at 12 weeks after STZ injection. AS-IV treatment (2.5, 5, 10 mg·kg^−1^·d^−1^) was started 2 weeks before STZ injection and lasted 14 weeks. NC, normal control rats; DN, STZ-induced diabetic rats; AL, DN rats treated with AS-IV (2.5 mg·kg^−1^·d^−1^); AM, DN rats treated with AS-IV (5 mg·kg^−1^·d^−1^); AH, DN rats treated with AS-IV (10 mg·kg^−1^·d^−1^).

**Figure 6:**
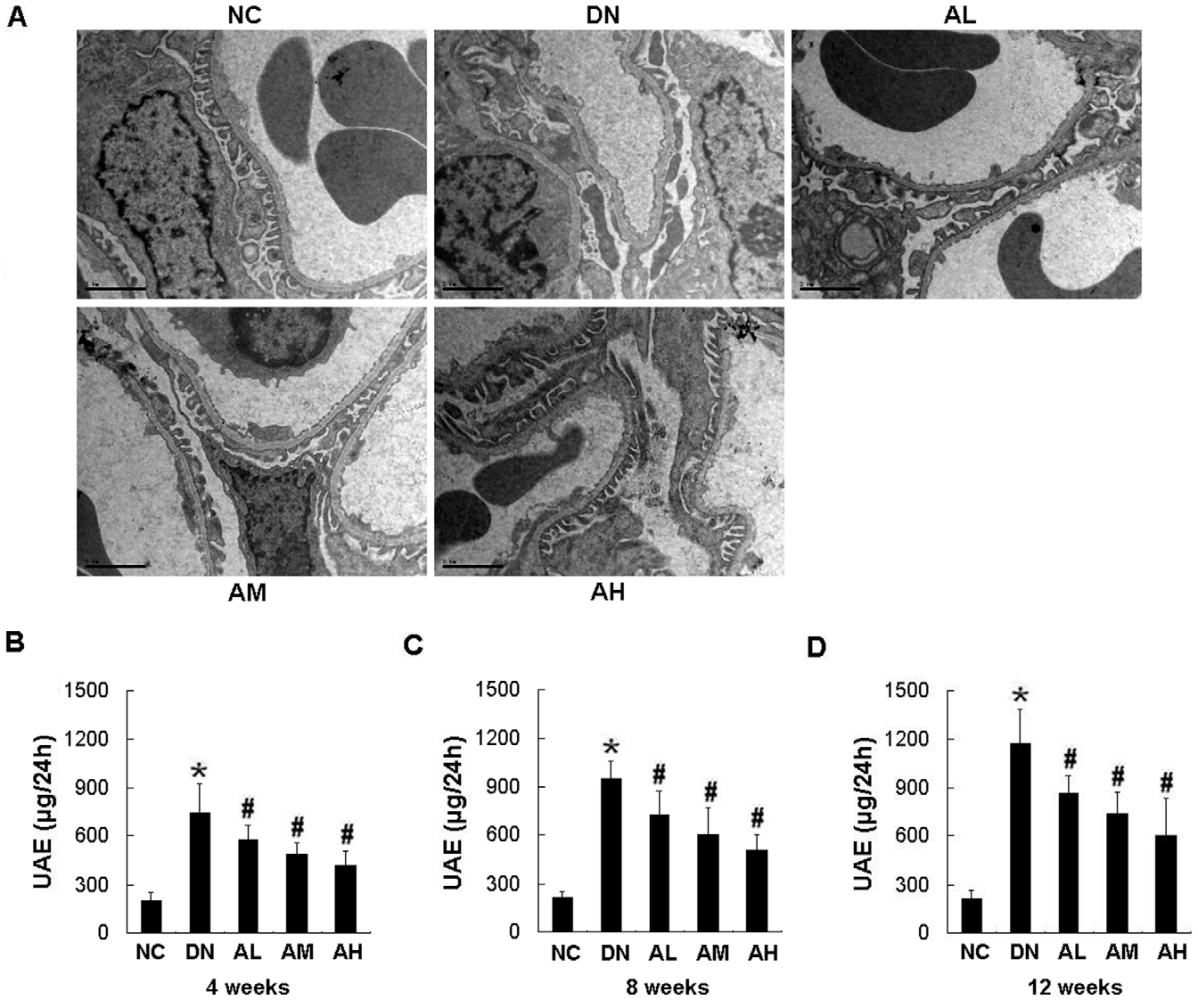
AS-IV dose-dependently ameliorated podocyte foot process effacement and reduced the albuminuria in diabetic rats. Effects of AS-IV on glomerular ultrastructure (A) and albuminuria in diabetic rats at 4 weeks (B), 8 weeks (C) and 12 weeks (D) after STZ injection. UAE, urinary albumin excretion. Results are expressed are the means ± SD (n = 8/each group). *P<0.05 vs NC. ^#^ *P*<0.05 vs DN.

**Figure 7:**
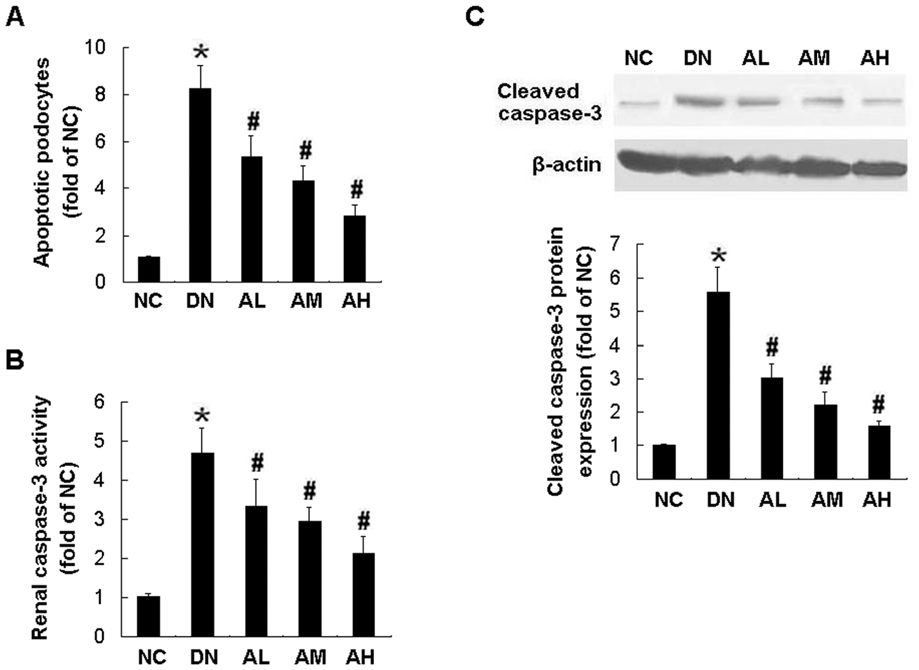
AS-IV dose-dependently prevented podocyte apoptosis, caspase-3 activation and overexpression in diabetic rats. Effects of AS-IV on podocyte apoptosis detected by TUNEL assay (A), caspase-3 activity (B) and cleaved caspase-3 protein expression (C) in diabetic rats at 12 weeks after STZ injection. Results are expressed are the means ± SD. *P<0.05 vs NC. ^#^ *P*<0.05 vs DN.

**Figure 8:**
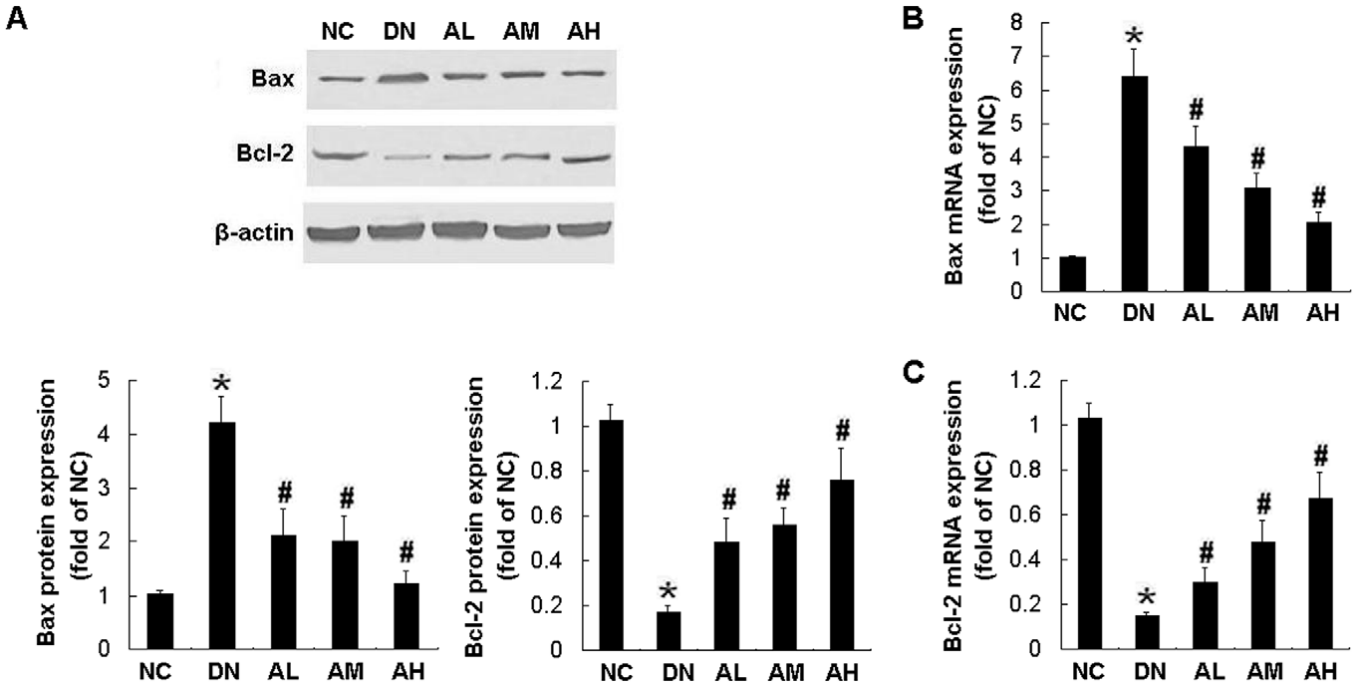
AS-IV restored the balance of Bax and Bcl-2 mRNA and protein expression in diabetic rats. Effects of AS-IV on protein expression of Bax and Bcl-2 (A), as well as mRNA expression of Bax (B) and Bcl-2 (C) in diabetic rats at 12 weeks after STZ injection. The protein expression of Bax and Bcl-2 was examined by Western blotting. The mRNA expression of Bax and Bcl-2 was examined by Real-time RT-PCR. Results are expressed are the means ± SD. *P<0.05 vs NC. ^#^ *P*<0.05 vs DN.

### Determination of ROS production and levels of oxidative stress markers

ROS generation in podocytes was measured with the fluorescent probe 2′,7′-dichlorodihydrofluorescin (DCF) diacetate as previously described [18]. Briefly, podocytes were incubated for 45 min at 37°C with 50 µmol/l DCF diacetate. The DCF fluorescence was determined in a multiwell fluorescence plate reader.

The oxidative stress markers, including activities of catalase (CAT) and superoxide dismutase (SOD), and content of malondialdehyde (MDA) in kidney cortex and cultured mouse podocytes were determined using the assay kits (Nanjing Jiancheng Bioengineering Institute, Nanjing, China), according to the manufacturer's instruction.

### Western blotting

Kidney cortex or cultured podocytes under different experimental conditions were lysed in lysis buffer with a sonicator. Crude lysates were centrifuged at 10,000 rpm for 5 min at 4°C. Total protein concentration from the resultant supernatant was determined by the bicinchoninic acid (BCA) protein assay (Pierce, Rockford, IL, USA). Reduced protein samples were separated by sodium dodecyl sulfate (SDS)/polyacrylamide gel electrophoresis, transferred to a polyvinylidene difluoride membrane (Immobilon-P, Millipore, Bedford, Mass, USA) by electroblotting. Membranes were incubated overnight at 4°C with the following primary antibodies: anti-Bax and anti-Bcl-2 antibodies (Santa Cruz Biotechnology, CA, USA), anti-cleaved caspase-3 antibody (Cell Signaling Technology, MA CA, USA). Negative controls were performed without primary antibody. After washing, the secondary antibody was added and incubated 1 h at room temperature and protein bands were visualized by ECL Plus (Amersham, Arlington Heights, IL, USA). The membranes were then stripped and re-probed with anti-β-actin antibody (Sigma, USA). Densitometric quantitation was performed using a Bio-Rad VersaDoc imaging system model 5000 with Bio-Rad Quantity One software. Protein expression was quantified as the ratio of specific band to β-actin.

### Real-time quantitative RT-PCR

Total RNA from kidney cortex or cultured podocytes was isolated by the Trizol procedure (Invitrogen, Carlsbad, CA, USA). Then, 1 µg of total RNA was reverse transcribed using the SuperScript RT kit from Invitrogen (Carlsbad, USA). Quantitative RT-PCR was performed using the ABI PRISM7900 Sequence Detection System (Applied Biosystems) with SYBR Green Master Mix and three-step standard cycling conditions and sequence-specific primers. Primer concentrations were determined by preliminary experiments that analyzed the optimal concentrations of each primer. The following sequence-specific oligonucleotide primers for mouse podocytes were used for PCRs: Bax sense GTTTCATCCAGGATCGAGCAG and anti-sense AGCTGAGCGAGTGTCTCCGGCG; Bcl-2 sense CTGAGTACCTGAACCGGCATC and anti-sense GAGCAGCGTCTTCAGAGACAG. The following sequence-specific oligonucleotide primers for rat kidney tissue were used for PCRs: Bax sense AGACACCTGAGCTGACCTTGGA and anti-sense CGCTCAGCTTCTTGGTGGAT; Bcl-2 sense GGGATGCCTTTGTGGAACTATATG and anti-sense CAGCCAGGAGAAATCAAACAGA. In order to confirm amplification specificity, the PCR products from each primer pair were subjected to a melting curve analysis and subsequent agarose gel electrophoresis. A control without cDNA was run in parallel with each assay. Each reaction was amplified in triplicate and ratio results were calculated based on the 2^−ΔΔCT^ method as described previously [19]. Relative mRNA levels were normalized to those of GAPDH.

### Statistical analysis

Statistics were conducted by SPSS 13.0 software. All data were expressed as means ± standard deviation (SD). The significance of differences among experimental groups was determined by ANOVA analysis. When a significant difference was detected, the data were further analyzed by Dunnett's multiple range test. A value of *P*<0.05 was considered statistically significant.

## Results

### AS-IV protected mouse podocytes from apoptosis induced by high glucose (HG)

The apoptosis of podocytes was examined by two different methods in vitro. As shown in Hoechst staining, exposure to HG for 24 h resulted in a significant podocyte apoptosis compared with NG control (Fig. 1A). MA had no effect on cell apoptosis. However, AS-IV protected against HG-induced podocyte apoptosis in a concentration-dependent manner, with the maximal effect achieved at a dose of 200 µg/ml (Fig. 1A). The effect of AS-IV on podocyte apoptosis was also determined by FACS after Annexin V-FITC and PI labeling. Likewise, AS-IV reduced the podocyte apoptosis at concentration of 50, 100 and 200 µg/ml, respectively, when compared with HG control (Fig. 1B). Taken together, AS-IV significantly inhibited HG-induced podocyte apoptosis.

### AS-IV reduced the HG-induced ROS production and oxidative stress in podocytes

ROS played an important role in the pathogenesis of podocyte apoptosis in DN, we investigated the effects of AS- IV on ROS generation in podocytes. Compared with incubation in NG, exposure to HG for 24 h significantly induced ROS production (*P*<0.05). Exposure to MA for 24 h did not change the ROS level in podocytes. However, HG-induced ROS production was partially reduced by AS-IV in a dose-dependent manner (Fig. 1C). Moreover, the concentration of MDA and the activities of superoxide anion radical-scavenging enzymes, SOD and CAT in cultured podocytes were presented in Figure 2. Exposure to HG for 24 h resulted in a remarkable increase in levels of MDA (Fig. 2A) and a dramatical reduction in activities of CAT (Fig. 2B) and SOD (Fig. 2C), which were prevented by AS-IV treatment in a concentration-dependent manner (Fig. 2 A–C). These results demonstrated that AS-IV treatment significantly decreased the amount of a product of lipid peroxidation while increased the activities of antioxidant enzymes in cultured podocytes. Therefore, AS- IV effectively inhibited HG-induced ROS generation and oxidative stress in HG-stimulated podocytes.

### AS-IV suppressed the caspase 3 activation and overexpression in HG-stimulated podocytes

Apoptosis was further verified by detection of caspase-3 activity and Western blot analysis of caspase-3 cleavage. Exposure of podocytes to AS-IV resulted in a substantial decrease in the caspase 3 activity and caspase cleavage. HG-induced caspase 3 activation was restored by AS- IV in a concentration-dependent manner (Fig. 1D). AS- IV also dose-dependently downregulated the protein expression of cleaved caspase-3 (Fig. 3A). These results indicated that the antiapoptotic effect of AS-IV on podocytes was caspase-3 dependent.

### AS-IV regulated the mRNA and protein expression of Bax and Bcl-2 in HG-stimulated podocytes

HG elevated Bax mRNA expression and decreased Bcl-2 mRNA expression, which was abrogated by AS-IV treatment in a dose-dependent manner evident at a dose as low as 50 µg/ml (Fig. 3, B and C). In contrast, MA had no effect on mRNA expression of Bax (Fig. 3B) and Bcl-2 (Fig. 3C). Consistent with these effects on mRNA expression, HG for 24 h increased the amount of Bax protein and decreased the amount of Bcl-2 protein compared with NG. MA did not affect protein production of Bax and Bcl-2 while AS-IV treatment partially restored the balance of Bax and Bcl-2 protein expression in podocytes under HG condition (*P*<0.05) (Fig. 4). These findings indicated that the antiapoptotic effect of AS-IV was associated with regulation of the balance of Bax and Bcl-2 mRNA and protein expression.

### In vivo studies: Effects of AS-IV on body weight and metabolic parameters in blood

To validate these findings in cultured immortalized mouse podocytes, we established rat model of DN induced by intraperitoneal injection of STZ to test the hypothesis that AS-IV prevents podocytes apoptosis in vivo. Blood glucose (BG) and glycated hemoglobin (HbA1c) levels were significantly increased in STZ-induced diabetic rats (*P*<0.05 vs. normal conrol rats). However, no differences in BG and HbA1c levels were observed between AS-IV treated and untreated STZ-induced diabetic rats (Table 1). In diabetic rats at the end of 12 weeks after STZ injection, body weight was decreased compared with normal rats and did not change compared with rats that pretreated with AS-IV(Table 2). Additionally, AS-IV did not change the levels of blood urea nitrogen (BUN), serum creatinine (SCr) and alanine aminotransferase (ALT) in rats (Table 1), indicating that AS-IV had no toxic side effects to the liver and renal function.

### AS-IV significantly ameliorated the renal histopathology, podocyte foot process effacement, albuminuria and oxidative stress in STZ-induced diabetic rats

Mesangial matrix expansion is considered a hallmark of diabetic nephropathy. At 12 weeks after STZ injection, the diabetic rats showed focal mesangial matrix expansion compared to normal control rats and pretreatment with AS-IV ameliorated mesangial expansion compared with the untreated STZ-induced diabetic rats (Fig. 5). At 12 weeks after STZ injection, observation of podocyte ultrastructure by electron microscopy revealed marked podocyte foot process effacement in diabetic rats, whereas the rats pretreated with AS-IV showed a marked reduction in podocyte foot process effacement. AS-IV dose-dependently ameliorated podocyte foot process effacement in diabetic rats (Fig. 6A). The STZ-induced diabetic rats developed severe albuminuria when compared with the nornmal control rats (*P*<0.05). Pretreatment with AS-IV significantly alleviated proteinuria in diabetic rats. This effect was dose-dependent, which becoming evident at a dose as low as as 2.5 mg·kg^−1^·d^−1^ (Fig. 6 B–D). AS-IV also decreased albuminuria in diabetic rats in a time-dependent manner, which was evident in as little as 4 weeks after STZ injection (Fig. 6B). AS-IV at dose of 10 mg·kg^−1^·d^−1^ reduced the albuminuria by 48% at 12 weeks after STZ injection, which reached the peak effect (Fig. 6D). Moreover, AS-IV dose-dependently inhibited the oxidative stress in diabetic rats through decreasing the MDA content and increasing the activities of antioxidant enzymes (CAT and SOD) (Table 2).

### AS-IV prevented podocyte apoptosis in vivo

To determine if AS-IV has a similar anti-apoptotic effect in vivo that we have demonstrated in vitro, we assessed podocyte apoptosis in diabetic rats by TUNEL staining. There was a significant decrease in apoptotic podocytes in kidney sections from STZ-induced diabetic rats pretreated with AS-IV. This antiapoptotic effect was dose-dependent, which was as little as 2.5 mg·kg^−1^·d^−1^ of AS-IV (AL group), and the maximal inhibition was achieved by 10 mg·kg^−1^·d^−1^ of AS-IV (AH group) (Fig. 7A).

### AS-IV inhibited the caspase-3 activation and overexpression in STZ-induced diabetic rats

The caspase-3 activity and cleavage in kidney cortex from STZ-induced diabetic rats were measured. Similar to the results from in vitro study, AS-IV dose-dependently reduced caspase-3 activation (Fig. 7B) and caspase-3 overexpression (Fig. 7C) in STZ-induced diabetic rats.

### AS-IV restored the balance of Bax and Bcl-2 mRNA and protein expression in STZ-induced diabetic rats

Hyperglycemia-induced apoptosis was associated with increased Bax expression and decreased Bcl-2 expression at protein (Fig. 8A) and mRNA (Fig. 8, B and C) levels, which were partially restored by AS-IV in a dose-dependent manner. These effects were dose-dependent, which were evident at a dose as low as 2.5 mg·kg^−1^·d^−1^ and reached the maximal effect at 10 mg·kg^−1^·d^−1^ of AS-IV(Fig. 8 A–C).

## Discussion

Our study is the first to demonstrate the preventive effect of AS-IV, a novel antioxidant, on glucose-induced podocyte apoptosis, which has potentially important clinical consequences. This anti-apoptotic effect was shown by three different types of apoptosis assays. AS-IV dose-dependently inhibited high glucose (HG)-induced podocyte apoptosis in vitro. The studies in STZ-induced diabetic rats further demonstrated that pretreatment with AS-IV significantly ameliorated the renal histopathology, podocyte foot process effacement, albuminuria and oxidative stress. Moreover, glucose-induced podocyte apoptosis was associated with upregulation of Bax expression and downregulation of Bcl-2 expression, as well as caspase-3 activation and overexpression, which were partially restored by AS-IV in a dose-dependent manner. Taken together, AS-IV prevented glucose-induced podocyte apoptosis in vitro and in vivo, and these novel findings provided new insights into the development of kidney-protective drug through directly targeting podocytes.

There is mounting evidence demonstrating that podocytes have a central role in the progression of glomerular diseases and these glomerulopathies therefore are considered as podocytopathies [1]. However, most of the available therapies for renal diseases are unspecific and offer partial cures at best because these treatment options do not primarily focus on podocytes but rather act systemically and thus cause side effects. Thus, there is urgent need to develop potential drug therapy for proteinuria and kidney disease through direct podocyte targeting [20]. Recently, much work has demonstrated that podocyte apoptosis plays an important role in the pathogenesis of proteinuria and glomerulosclerosis in DN [21]–[24]. Experimental and clinical studies have shown that a decrease in podocyte number due to apoptosis or detachment leads to proteinuria in DN [3], [4], [25]. Therefore, preventing or inhibiting podocyte apoptosis should become an obvious and promising therapeutic target for treatment of DN. However, there are no current interventions specifically preventing podocyte apoptosis in DN.

Interestingly, the first major finding in this study was that AS-IV, one of the major and active components of the Astragalus membranaceus (Fisch) Bge, significantly prevented podocytes from apoptosis both in vitro and in vivo. We performed Hoechst staining, FACS after Annexin V-FITC and PI labeling, as well as TUNEL assay to exactly detect the podocyte apoptosis. Our previous study has verified that these methods are very accurate and reliable [26]. In the present study, HG induced ROS generation and podocyte apoptosis, which consisted with previous studies [7], [10]. This apoptotic effect was not due to an osmotic effect of HG because an equivalent concentration of D-mannitol did not cause the above result. However, AS-IV effectively inhibited the HG-induced podocyte apoptosis in a dose-dependent manner and this effect was evident at a dose as low as 50 µg/ml. The maximal inhibition of podocytes apoptosis by AS-IV was obtained at a dose of 200 µg/ml. Moreover, AS-IV reduced HG-induced ROS production in a dose-dependent manner. We also examined the effects of AS-IV on the content of MDA and the activities of antioxidant enzymes, CAT and SOD, in mouse podocytes cultured in HG. There was an increase in MDA level, as well as a decrease in CAT and SOD activities in podocytes exposed to HG, which was ameliorated by AS-IV treatment in a dose-dependent manner. Thus, AS-IV affected the oxidant-antioxidant balance in mouse podocytes. In vivo study further confirmed that pretreatment with AS-IV also dose-dependently reduced podocyte apoptosis. Podocyte apoptosis is an early glomerular phenotype that leads to progressive podocyte depletion and albuminuria, and oxidative stress played a critical role in the pathogenesis of podocyte apoptosis in DN [7], [10]. AS-IV has been found to have antioxidant effects both in vivo and in vitro [14]. In the present study, we also demonstrated that AS-IV reduced oxidative stress both in cultured podocytes and in kidney cortex from STZ-induced diabetic rats. The activities of antioxidant enzymes (CAT, SOD) were significantly increased and the MDA content decreased in AS-IV treated rats when compared with those in the untreated STZ-induced diabetic rats, suggesting a significant antioxidant effect of AS-IV. Furthermore, AS-IV ameliorated the albuminuria, renal histopathology and podocyte foot process effacement in diabetic rats. On the basis of these findings, we reasoned that AS-IV might improve podocyte survival, preserve podocyte number and reduce the albuminuria, therefore ultimately delay the progression of DN. This protective effect was partly attributed to its anti-oxidant action.

To explore the mechanisms underlying the antiapoptotic action of AS-IV, we examined the levels of Bax (proapoptotic) and Bcl-2 (antiapoptotic) proteins. It has been shown that Bax mediates podocyte apoptosis induced by TGF-β [8]. Decreased expression of Bcl-2 in podocytes is associated with progressive glomerular injury and clinical indices of poor renal prognosis in human IgA nephropathy [27]. The study in STZ-induced diabetic rats has demonstrated the increased expression of Bax and decreased Bcl-2 expression in renal glomeruli detected by immunohistochemical staining [23], [28]. In the present study, we further determined the mRNA and protein expression of Bax and Bcl-2 by real-time quantitative RT-PCR and Western blotting. We observed similar alterations in Bax and Bcl-2 expression in response to hyperglycemia. Our study showed that glucose induced apoptosis in vitro and in vivo, accompanied by an increase in Bax expression and a decrease in Bcl-2 expression, which were prevented by AS-IV treatment in a dose-dependent manner. Thus, AS-IV protects against podocyte apoptosis partly by restoring the balance of Bax and Bcl-2 proteins.

We further investigated the effects of AS-IV on the key downstream effector of apoptosis, caspase-3 activity and cleavage. Caspase-3 is an effector caspase and involved in many forms of apoptosis [29]. Activation of caspase-3 is the important determinant of apoptosis. Caspase-3 operates as the key effector enzyme in cell death through receptor-mediated (Fas/FasL) or mitochondria-dependent (Bax/Bcl-2)-induced apoptosis. In this study, caspase-3 acitivity and expression was increased both in HG-stimulated podocytes and in kidney cortex from diabetic rats, which consisted with the previous studies [7], [28]. AS-IV dose-dependently reduced caspase-3 activation and overexpression in vitro and in vivo. More importantly, the inhibitory effect of AS-IV on podocyte apoptosis paralleled the reduction in caspase-3 activation and overexpression. Therefore, the antiapoptotic effect of AS-IV on podocytes is caspase-3 dependent.

The above results indicate that AS-IV, a novel antioxidant, prevents glucose- induced podocyte apoptosis in vitro and in vivo, which is associated with decreasing Bax expression and inhibiting caspase-3 activation while increasing Bcl-2 expression.

Finally, we examined the blood glucose and HbA1c levels in rats. AS-IV exhibited no effects on the levels of blood glucose and HbA1c, thus the preventive effects of AS-IV on podocyte apoptosis and albuminuria were independent of the blood glucose levels. Moreover, we measured the levels of blood urea nitrogen, creatinine and alanine aminotransferase and tested the safety of AS-IV in rats. AS-IV had no toxic side effects to the liver and renal function in these rats, indicating that AS-IV may become a safe and effective drug for treatment of DN. In our preliminary study in rats, we chose the doses of AS-IV at 2.5, 5, 10 mg·kg^−1^·d^−1^, and we found that these doses could effectively reduce proteinuria and did not cause any toxicity. So these doses were chosen in our study.

In addition, a recent study has shown that AS-IV can reduce blood pressure in fructose-fed rats [30]. Therefore, We cannot exclude the possibility that some effects of AS-IV on podocyte's function may at least in part be induced by AS-IV mediated decrease of the blood pressure. These effects need further investigation.

There is growing evidence of gender-specific aspects in podocyte biology and renal diseases. Sex hormones may mediate the effects of gender on chronic renal disease. Several studies have demonstrated that aging male rats develop proteinuria and glomerulosclerosis, whereas female and estrogen-treated male rats are resistant to disease progression [31]–[33]. Data from meta-analysis studies indicate that men exhibit a more rapid age-related decline in renal function than women and several renal diseases appear to be gender-dependent [34]. Furthermore, recent report demonstrates that estrogen receptor alpha expression in podocytes protects against apoptosis in vitro and in vivo [35]. In this study, severe hyperglycemia and albuminuria, as well as increased apoptosis were developed in male rats with DN. However, whether gender plays a role in promoting podocyte apoptosis and progression of diabetic kidney diseases needs further study.

In summary, our results demonstrate for the first time that AS-IV acts directly on podocytes. AS-IV, a novel antioxidant, prevents podocyte apoptosis and reduces albuminuria in DN partly through decreasing Bax expression and inhibiting caspase-3 activation while increasing Bcl-2 expression. These findings strengthen the therapeutic rationale for AS-IV in the treatment of DN and also provide new insights into the development of kidney-protective drug through directly targeting podocytes.
